# Development of an Ingestible Expandable Capsule for Weight Loss

**DOI:** 10.3390/ijerph192416821

**Published:** 2022-12-15

**Authors:** Xingyu Liu, Yeung Wu, Chang Liu, Kaiqi Chen, Hans Gregersen

**Affiliations:** 1 Graduate School, Chongqing Normal University, Chongqing 401331, China; 2 Department of Surgery, The Chinese University of Hong Kong, Shatin, Hong Kong 999077, China; 3 GIOME, California Medical Innovations Institute, San Diego, CA 92121, USA

**Keywords:** obesity, overweight, weight loss, expandable capsule, EndoXpand

## Abstract

Obesity has grown to epidemic proportions with 2.1 billion people being overweight worldwide. A food-grade expandable capsule named EndoXpand for the treatment of overweight people was designed and developed in this study. EndoXpand consists of an inner expandable material (core), an embracing membrane, and a gelatin capsule shell. It is designed to occupy volume in the stomach and reduce hunger sensation. The occupied volume is changeable over time, dependent on the number of ingested capsules and their degradation time. This will avoid gastric accommodation to constant volume devices as seen in the use of intragastric balloons. Several materials were tested. Collagen casing was selected as the membrane and corn silk was used to tie the membrane. Dried black fungus (*Auricularia auricula*) was the biological material that expanded most. However, synthesized cellulose-based hydrogel expanded more and was chosen as the optimal expandable core material. The hydrogel-based EndoXpand expanded 72 times after soaking in an acidic environment for 80 min. The corn silk ligations weakened and broke after 3 h. This resulted in release of the expanded material that was designed to easily pass the pylorus and travel down the intestine for digestion or excretion. In conclusion, this study provides design and *in vitro* proof-of-technology data for a potential groundbreaking approach. Further studies are needed in animal models and human phase I studies.

## 1. Introduction

According to the World Health Organization, obesity is a growing global health concern. Persons with BMI of 30 or more are considered obese and persons with BMI equal to or more than 25 are considered overweight [1]. The estimated number of overweight and obese adults worldwide is 2.1 billion [2]. The global COVID-19 pandemic may also have accelerated the increase in obesity prevalence. Being overweight is associated with increased risk of diseases including arterial hypertension, dyslipidemia, type 2 diabetes mellitus, and coronary heart disease [3]. Weight loss, even as low as 10%, will significantly decrease comorbidities [4].

The increased prevalence of overweight and obesity has led to numerous weight loss therapies, each with advantages and disadvantages. Diet is the most common but often fails long-term. Anti-obesity pharmacotherapies such as orlistat, lorcaserin, phentermine, naltrexone, and liraglutide [5] are relatively ineffective and have side effects such as nervousness, restlessness, and excitability [6]. Persons with morbid obesity (BMI > 35 kg/m^2^) or moderate obesity complicated by comorbid conditions, that are not responsive to non-surgical approaches, may benefit from bariatric surgery that has been demonstrated to be the most effective long-term treatment. Bariatric surgery changes the geometry of the stomach, i.e., reduces the effective size. However, it is invasive, irreversible, and may have complications. Furthermore, it is expensive, with a cost of between $15,000 and $25,000 for each operation [7]. Intra-gastric balloons (IGBs) are an alternative to bariatric surgery [6,8] where a silicone balloon is placed endoscopically in the stomach and filled with air or saline. Usually, 10% weight loss can be achieved within 6 months. However, usually after 6 months, no further weight loss is achieved. The mechanism of action is that the balloon fills the stomach, i.e., reduces the stomach capacity, which induces satiety with less food intake. However, in the first seven to ten days after balloon insertion, discomfort, nausea, and vomiting are very common [9]. Considering the relatively sparse weight loss with IGBs, we believe that the lack of efficacy is due to stomach accommodation to the constant IGB volume. The stomach is used to being empty from time to time and changes volume almost constantly during meals, snacks, and when drinking fluids. We believe that accommodation can be prevented by changing the occupied volume of the stomach. Needless to say, that is not possible with IGBs since endoscopic procedures are required to inflate them and to change the volume.

In this study, we introduce a new design and concept for weight loss treatment based on expandable materials. *In vitro* testing of biomaterials and devices is needed before any testing can go on in animals and humans. Prior to clinical investigation, *in vitro* testing provides an easy, fast, and economic evaluation of specific material properties, such as morphological characteristics, chemical compositions, surface tension, mechanical property and biocompatibility [10]. Key parameters of biomaterials determined by *in vitro* testing constitute important indices for evaluation of validity, applicability, and safety of the tested biomaterial *in vitro* [11]. Therefore, establishment of credible *in vitro* test techniques is required. The test setup must simulate repeatable parameters and responses imitating biological effects of the patient after ingesting the material.

To overcome the limitations of traditional therapies, we introduce a biological expandable capsule design named EndoXpand based on bionic considerations. The goal was to design and develop a capsule-based therapy that will result in 0.5 kg weight loss per week. The design is simple and economic, and the capsule size is suitable for swallowing. Based on the design, we believe that EndoXpand will be safe, low cost, easy to administer by ingestion, and offer more efficient weight loss treatment schemes thanks to the flexible administration process. Long-term, we expect to provide over-the-counter weight-loss capsules for obese and overweight people that are relatively free from side effects and easy to self-administer. Upon ingestion of a variable numbers of capsules, EndoXpand will occupy space in the stomach and change the occupied volume readily to avoid the accommodation effect. The design facilitates fast swelling speed and a high swelling ratio of the EndoXpand capsules that consist of food grade materials. EndoXpand possesses potential advantages over conventional weight loss therapies due to the high water content and softness, which sustain gastric retention and minimize the risk of potential complications such as inflammation of the stomach wall. EndoXpand can be self-administered and hence will eliminate hospitalization, rehabilitation, and complications associated with endoscopic or surgical procedures, and effectively avoid the long-term side effects from drugs. To validate the proposed protocol, extensive evaluation of biomaterials was done to determine the optimal material candidates for the expandable core and for the embracing membrane. In this study, we designed the EndoXpand capsule and tested it *in vitro* under physiological conditions. The aim was to lay the foundation for future testing on animals and humans in clinical trials.

## 2. Materials and Methods

### 2.1. Design of the EndoXpand Prototype

**Figure 1:**
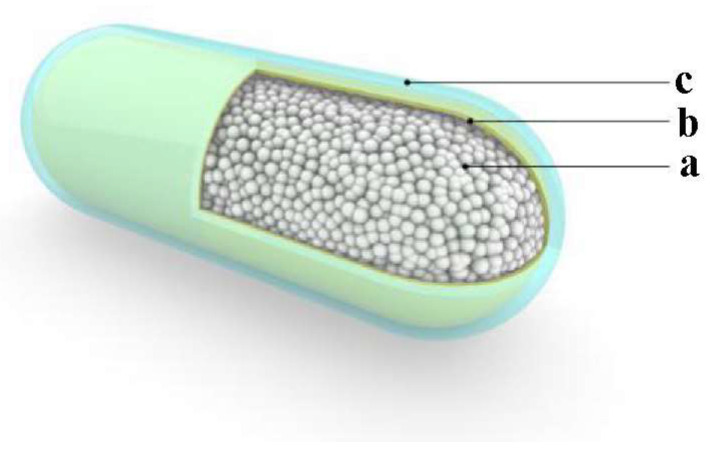
Schematic diagram of the EndoXpand prototype: a is the core expandable material; b is the outer membrane to encapsulate the expandable materials; c is a 000 standard size gelatin capsule shell. The dimensions are: external diameter: 9.55 ± 0.135 mm; length: 26.1 ± 0.3 mm; average wall thickness: 0.145 mm; empty prototype core volume capacity: 1.37 mL; outer membrane wall thickness: 0.035 mm; and gelatin capsule shell wall thickness: 0.11 mm.

The basic design of the expandable biological capsule is illustrated in Figure 1. It is designed as a 25 mm long and 10 mm diameter shell with rounded ends and smooth non-sticky surface. The dimensions were chosen in consideration of the maximum size of object an average person is likely to swallow. It can be made smaller for persons who cannot swallow large capsules. The ingestible capsule consists of three layers. The outermost surface layer is a standard 000 gelatin capsule with 0.11 mm thickness. The middle layer is the outer membrane, which functions to keep the expandable content of the innermost core together.

The design concept is based on the fact that the gelatin shell dissolves within minutes in the acidic fluid of the stomach. The stomach fluid will penetrate the outer membrane and reach the expandable material in the core. Drinking a glass of water immediately before, during, or after ingesting the capsule facilitates fast swelling. EndoXpand must swell to a diameter that prevents passage through the pylorus (limit diameter 19 mm) to ensure that the expanded capsule remains in the stomach for the desired time. Other requirements were that (1) the capsule will expand into a spherical shape and reach 50% of maximum expansion within 15 min and full expansion in 60 min, (2) that the final volume of a single capsule will be between 50–200 mL, and (3) that it will break down/dissolve within controllable time, e.g., within 3, 5, or 24 h. The breakdown of the outer membrane will release the core material, i.e., smaller core particles can pass into the intestines through pylorus without obstructing the intestine and be digested or excreted.

### 2.2. Expandable Materials of the EndoXpand Prototypes

Expandable food-grade materials can be natural or synthetic. Our first approach was to use natural materials commonly known to expand considerably in water. Natural edible dried black fungus (*Auricularia auricula*), chia seeds, konjac root, and kelp were purchased. Granular black fungus was produced from the dried intact fungus in a standardized way using 3D printed molds with compressors by polylactic acid (Lian Dong San Wei Technology Co., Ltd., Fanchang, China). The granular black fungus was inserted into cylinders that fitted exactly the size of the 000/size capsule. Furthermore, cellulose-based hydrogel bulking agents were synthesized from food-grade sodium carboxymethyl cellulose (304 mpa.s 25 °C, DS 0.92, 8.98 wt% sodium, food grade), hydroxyethyl cellulose (M.S 2, 3000 mpa.s 25 °C, food grade) and citric acid purchased from WanBang Chemical Co., Ltd., Dongguan, China.

### 2.3. Cellulose-Based Hydrogel Bulking Agents Synthesis

Hydrogel bulking agent materials were synthesized in deionized water, sodium carboxymethyl cellulose (NaCMC), and hydroxyethyl cellulose (HEC) using citric acid as a crosslinking agent according to the following procedure. First, a total polymer concentration of 2% by weight of H_2_O was mixed with NaCMC and HEC with weight ratio 5:1. It was dissolved in deionized water by stirring at 300 revolutions/min at 30 °C for 20 h until a clear mixture was obtained. Finally, citric acid (37.5% *w*/*w* polymer) was added to the clear mixture. The final solution was poured into a glass mold. All materials were air-dried at 35 °C for 24 h to remove absorbed water and then kept on a heat plate at 75 °C for 1 h for the crosslinking reaction [12].

### 2.4. Outer Membrane of the EndoXpand Prototypes

Food grade materials were used in the present study. Tubular membranes are often used in food processing. As the initial approach, tubular hog casing and collagen casing were purchased.

### 2.5. Closure of the Outer Membranes

To close the ends of the tubular membrane with a biological material, natural plant fibers with small diameters, that are bendable and degradable, are the ideal choice. After reviewing the properties of a wealth of plant fibers, we decided to use 8–10 fibers of corn silk that were twisted into a thicker string.

### 2.6. Swelling Ratio of Expandable Materials

Equilibrium swelling measurements for all natural and synthetic expandable materials were carried out using a Sartorius analytical balance (Model BP121S, Sartorius, precision 0.1 mg). The swelling ratio was measured by weighing expandable materials before and after their immersion in simulated gastric fluid (SGF) at different time intervals. The swelling ratio was defined as:

SR = (*W_f_* − *W_tea_* − *W_i_*)/*W_i_* (1)

where SR is the swelling ratio; *W_f_* is the final weight of the expandable materials; *W_tea_* is the weight of the dried teabag after immersion in distilled water for 20 min; and *W_i_* is the initial weight of the dried materials.

### 2.7. Degradation of Expandable Materials, Outer Membranes and Corn Silk

As the first approach, the expandable capsules were designed to stay in an environment simulating the stomach for minimum 3–4 h. To study the degradation of the expandable materials and outer membranes, natural expandable materials and outer membranes were dried in the oven at 50 °C to obtain the initial dried weight (*W*_1_). The natural dried expandable materials and outer membranes were submerged in SGF at 37 °C for one day and were drained to remove excess water. Further, they were dried in a 50 °C oven until the weights were constant and recorded as the final dried weight (*W*_2_). Finally, the degradation of the expandable materials and outer membranes was defined as:

Degradation (%) = (*W*_1_ − *W*_2_)/*W*_2_ × 100% (2)

Mechanical Testing System (Mach-1, Biomomentum Inc., Quebec, Canada) was used to obtain stress-stretch curves of corn silk fibers. Using this system, the corn silk fibers were mounted on the grips. We conducted a stress-stretch test of corn silk samples with speed 0.15 mm/s. The average diameter of a corn silk sample was 0.125 mm. The corn silk samples were submerged in SGF and deionized water (pH 7) at 37 °C for 4 h. We took fibers out every hour and hung them on the shelf to investigate the degradation of the corn silk. An 8 g metal weight was hung under each corn silk to check whether the fiber would break.

### 2.8. Ultrasonographic Study of Expanded EndoXpand In Vitro

A standard ultrasound scanner for studying internal organs in veterinary clinics was used to visualize EndoXpand in a fluid-filled organ bath. The ultrasound head was held 5–10 cm from the EndoXpand to simulate intragastric conditions.

### 2.9. Statistical Analysis

All experiments and measurements were carried out at least three times. Results were expressed as the averages of paralleled independent trials. Analyses of standard deviation, ANOVA, and significant difference were done using SPSS 19.0 and Excel software. *p* < 0.05 was considered statistically significant.

## 3. Results

### 3.1. Swelling Ratio of Expandable Materials

**Figure 2:**
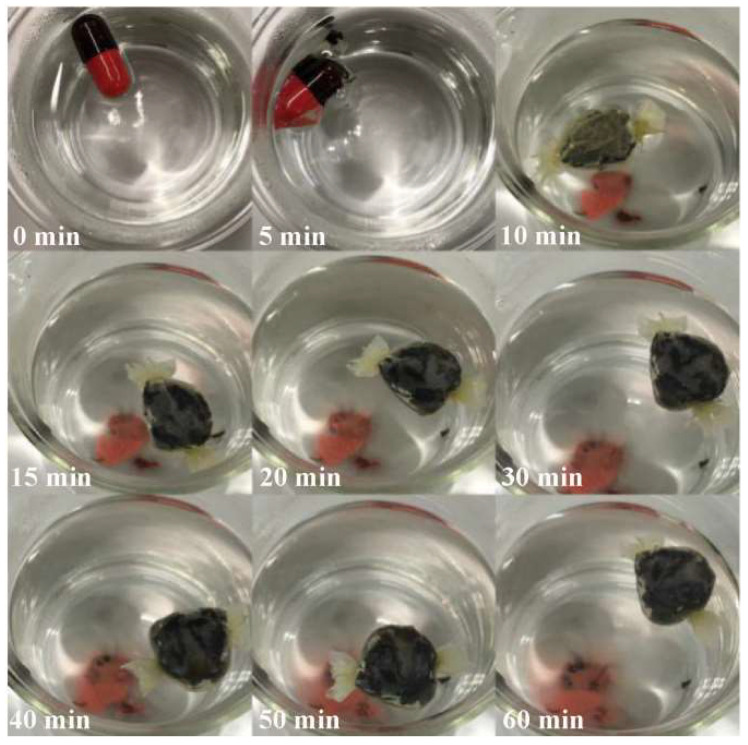
Black fungus inside the casing and the gelatin capsule after shaping in the 3D printed molds. The photos are from taken at 0, 5, 10, 15, 20, 30, 40, 50, and 60 min. The final expanded size was 2.5 cm in diameter.

**Figure 3:**
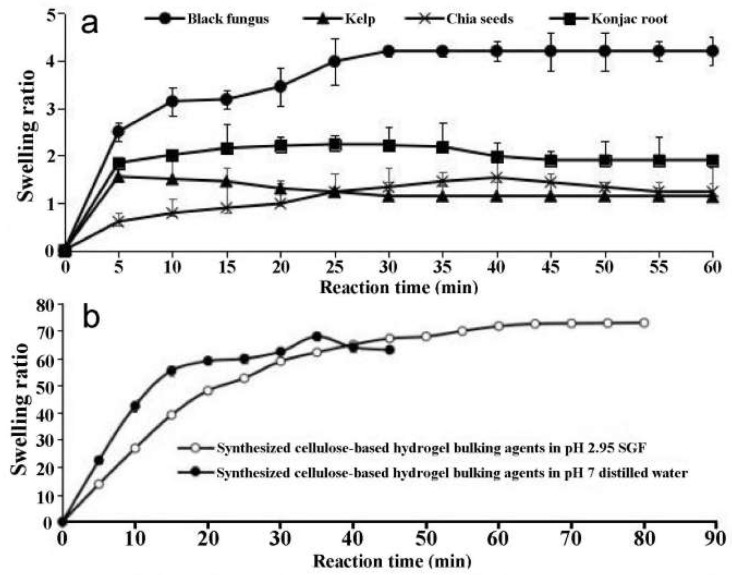
Swelling as function of time of natural and synthesized expandable materials. (**a**) The swelling ratio of four natural expandable materials: dried black fungus, chia seeds, konjac root, and kelp for 60 min in pH 2.95 simulated gastric fluid. (**b**) The swelling ratio of synthesized cellulose-based hydrogel bulking agents in pH 7 distilled water and pH 2.95 simulated gastric fluid.

Figure 2 shows an example of expansion of a capsule with intact black fungus during a bench test. The final diameter of the spherical EndoXpand was approximately 2.5 cm. Intact black fungus had the highest swelling ratio, approximately 4.2 when compared with chia seeds, konjac root, and kelp (Figure 3a). For most materials, more than half of the final swelling was obtained within 5 min. The granular form had a ratio of 11.98 in the first 20 min, reaching maximum ratio of 12.6 in SGF, whereas intact black fungus had a maximal ratio of 6.2 in SGF. Since double the amount of granular fungus could be loaded into the capsule and granular fungus swelled double as much as intact fungus, granular fungus is four times better than intact fungus.

The swelling ratio of cellulose-based hydrogel bulking agents (Figure 3b) was much higher that the swelling ratios of the natural materials (Figure 3a). Synthetic cellulose-based hydrogel bulking agents in SGF swelled to weight ratio 48, 60, and 72 after 20, 30, and 80 min, respectively (Figure 3b). The density of the synthesized cellulose-based hydrogel bulking agents was 1.06 g/mL, i.e., in volume, synthesized cellulose-based hydrogel bulking agents in pH 2.95 SGF swelled to ratio 45, 57, and 68 in 20, 30, and 80 min, respectively. Half of the swelling was obtained in 15 min or less. Furthermore, for each of the synthesized cellulose-based hydrogel bulking agents in pH 2.95 SGF, the swelling ratio did not change further from 60 to 80 min, ending at values around 71–72. Hence, the synthesized cellulose-based hydrogel bulking agents reached maximum in 60 min.

### 3.2. Degradation of Expandable Materials

**Figure 4:**
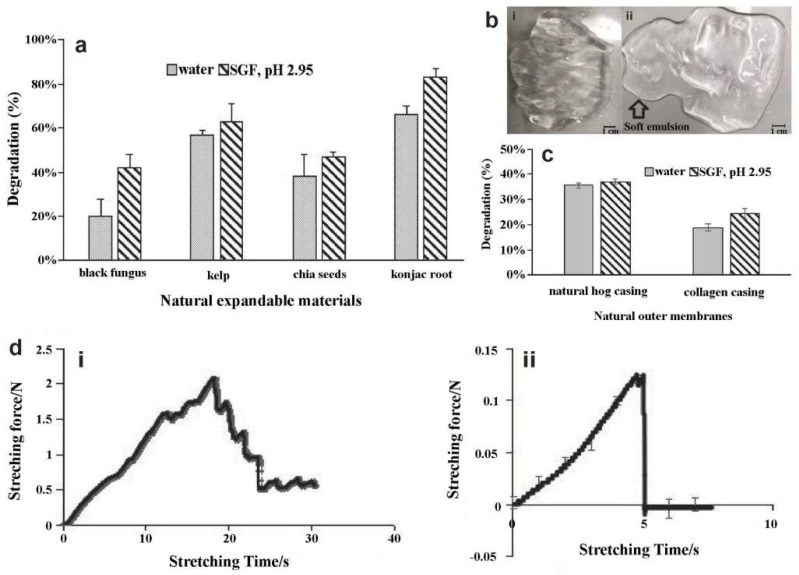
Degradation of EndoXpand capsules. (**a**) The degradation of four natural expandable materials (black fungus, kelp, chia seeds, and konjac root) upon submerging in pH 2.95 simulated gastric fluid (SGF) and pH 7 deionized water for 24 h. For each material, difference was found between the degradation in water and SGF (*p* < 0.05). (**b**) Testing for 3D synthesized cellulose-based hydrogel bulking agents. (i) Hydrogel bulking agents immersed after 80 min in pH 2.95 SGF and maintained its 3D elastic gel structure. (ii) Hydrogel bulking agents immersed after 35 min in pH 7 distilled water, partially degrade (almost 50% degradation) and become a soft emulsion. (**c**) Degradation of the two membrane materials in pH 2.95 SGF and pH 7 deionized water at 37 °C after 10 h submersion (*p* < 0.05 for each material). (**d**) Stretch−force curves for corn silk fibers (i) without submersion in SGF and (ii) after submersion in SGF for 3 h. The force at the point of breakage is reduced by more than a factor 10. *n* = 6.

The water degradation of the black fungus was approximately 20%, which was 2.6, 1.75, and 3.5 times less than dried kelp, chia seeds, and konjac root, respectively (Figure 4a). Furthermore, the SGF degradation of black fungus (42%) was the lowest among the natural materials tested.

The synthesized cellulose-based hydrogel bulking agents still maintained the 3D elastic structure after 80 min in pH 2.95 SGF (Figure 3b and Figure 4bi). In pH 7 distilled water, the synthesized cellulose-based hydrogel bulking agents partially degraded after 35 min and became soft emulsions (Figure 3b and Figure 4bii). They could no longer be regarded as intact 3D gels. Hence, the equilibrium swelling measurement was stopped.

### 3.3. Degradation of the Membrane and Corn Silk

Both hog and collagen casings partially degraded in pH 2.95 SGF and in pH 7 deionized water at 37 °C (Figure 4c). However, both materials still appeared strong and would be unlikely to break and release the core material even after 24 h.

**Figure 5:**
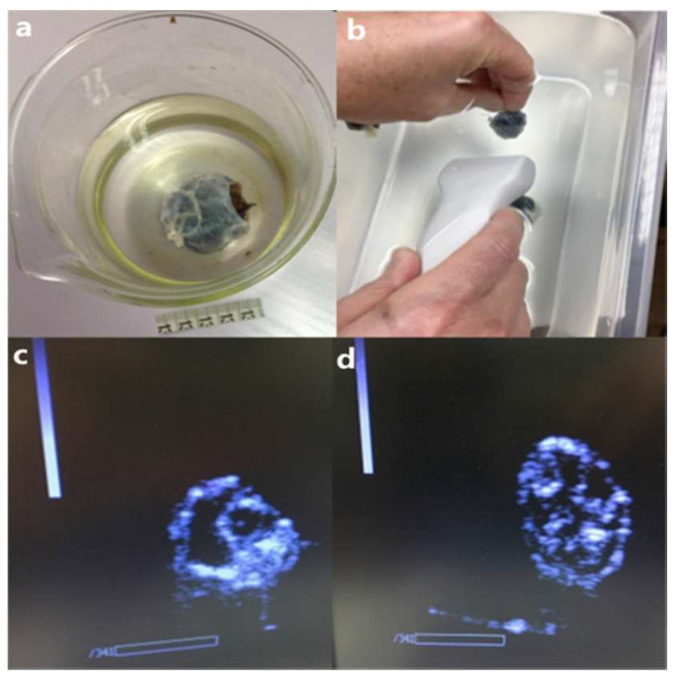
Ultrasonic examination of EndoXpand. (**a**) Intact black fungus covered by the outer membrane of casing. The corn silk ligature at one end had at this point degraded and broke. This released the black fungus pieces to the surrounding fluid. Since the individual pieces are of much smaller size, they will be able to pass the pylorus in humans without causing intestinal obstruction. (**b**) The testing environment for ultrasound experiments. (**c**) Ultrasound images of EndoXpand capsules with intact black fungus and granular black fungus (**d**).

Corn silk fibers with high flexibility for closure of the two ends of the tubular membranes could sustain forces up to 1.51 N (Figure 4di). The corn silk was significantly weakened after 3 h submersion in SGF (Figure 4dii). The 8 g weight broke the fibers after 3 h, which was confirmed after 4 h of submersion. These results demonstrate the weakening in SGF, i.e., it withstood less than 0.12 N force. Since corn silk ligations degrade significantly in SGF, it will release the expandable core materials from the outer membrane after 3–4 h. Figure 5a shows an example from a bench test where the corn silk at one end broke and the intact black fungus in the core is starting to escape.

### 3.4. Ultrasound Detection of Endoxpand

To prepare for future studies in animals and humans, we studied whether ultrasonography would be able to visualize EndoXpand with intact fungus inside the stomach. EndoXpand was clearly visible in the *in vitro* test setup (Figure 5b–d).

## 4. Discussion

In brief, we have proposed the design of an ingestible capsule for expansion in the stomach as a treatment for overweight people. In this study, we tested several biological and synthetic materials and provided bench testing results for further translation to animal and human experiments.

### 4.1. High-Ratio Swelling of Synthesized Cellulose-Based Hydrogel Bulking Agents

Synthesized cellulose-based hydrogel bulking agents were obtained through synthesis of NaCMC, HEC, and citric acid. They showed an impressive maximum swelling ratio of 72. HEC is necessary to promote intermolecular instead of intramolecular crosslinking. Poor crosslinking efficiency is observed when only NaCMC is used. This is due to the high degree of substitution of hydroxyl group at the C6 most reactive position, and electrostatic repulsion between polyelectrolyte chains [12]. Furthermore, it is known that effective swelling of cellulose-based hydrogels requires a crosslinked network that can be obtained chemically, for example by esterification. In recent studies, citric acid was used as crosslinking agent in various cellulose derivative systems. The reaction is based on anhydride intermediate formation in two main stages [13]. The esterification reaction of the first cyclic anhydride exposed a new carboxylic acid (-COOH) unit in citric acid, allowing the attachment of carboxylic acid moiety to cellulose hydroxyl (-OH) group. Further reaction through esterification with another cellulosic hydroxyl group (-OH) will produce crosslink between cellulose chains. Therefore, further reaction through esterification with another cellulosic hydroxyl group will produce crosslink between cellulose chains.

### 4.2. pH-Responsive Synthesized Cellulose-Based Hydrogel Bulking Agents

NaCMC is a derivative of cellulose in which -CH2COOH functionalization takes place by the replacement of -OH groups from the cellulose backbone [14]. Firstly, in highly acidic aqueous medium (pH < 3.5), the NaCMC polymer chain was poorly ionized. When the pH increased gradually, the negatively charged species (-OCH2COO-) increased significantly and complete dissociation occurred at neutral pH = 7. Consequently, the intra- and intermolecular repulsion interactions increased. At this stage, the intermolecular repulsive forces acted against the intramolecular repulsive interaction and expansion of the NaCMC chain, causing deprotonation and formation of dissociated carboxymethyl species [15]. Therefore, according to Figure 4b, synthesized cellulose-based hydrogel bulking agents are pH sensitive. They can keep the same shape of the synthesized cellulose-based hydrogel bulking agents after gelation onset in SGF (pH = 2.95) and can degrade in non-acidic distilled water at pH = 7.

### 4.3. Deswelling Characteristics of the EndoXpand Capsules

For the degradation of natural outer membranes in SGF, we observed that the degradation time of both collagen and hog casings was 24 h or longer. However, we aimed at faster degradation in these initial studies. Corn silk ligations will loosen after 3 h submersion in SGF facilitating release of the core material to the surrounding fluid. We believe that fibers already exist or can be developed that will break down in a controlled time at certain pH values. For example, it will be expedient if fibers break down much faster at alkaline pH.

### 4.4. Safety Issues

As for any food grade product, risk assessment and safety are important. The most serious risk is that the capsule expands in the intestines rather than in the stomach. This may cause intestinal obstruction and pain. This has been considered in our design and can be further mitigated in several ways. For example, expansion is facilitated in the stomach if sufficient amounts of fluid are ingested at the same time. Furthermore, ingestion of just a small amount of nutrient (could potentially be placed in the capsule) will delay gastric emptying, which generally occurs 30–90 min after ingestion [16]. Hence, it will provide time for EndoXpand capsules to expand before gastric emptying takes place. Moreover, membranes and fibers can be engineered to break down faster in alkaline environments, meaning that breakdown will occur fast if expansion occur in the intestines. In addition, on-demand shrinkage hydrogel outer membrane will be considered in our future studies. Another way to mitigate, and as a rescue strategy, is to ingest chelating agents in tolerable oral dosages to break down the crosslinked network of the hydrogel outer membrane. On-demand shrinkage of EndoXpand capsules may not only be used as a rescue tool for potential complications but may also serve as an approach to control gastric retention time [17].

It is well known that IGBs irritate gastric mucosa [18]. This side effect may also apply to EndoXpand. However, EndoXpand is much smaller than IGBs and has a food-grade surface. This needs to be studied in animal experiments.

The outer membrane (i.e., hog casing, collagen casing) was edible food. Furthermore, all prepared natural expandable materials, i.e., black fungus, chia seeds, konjac root, and kelp are edible food. For the synthetic expandable materials to achieve the goal of 0.5 kg weight loss per week, daily intake of 8.34 g of hydrogel bulking agent is required. This contains a daily intake of 2 g of NaCMC, 0.417 g of HEC, and 0.94 g of citric acid, which is regarded safe.

NaCMC ingested per day contains 0.173 g of sodium. Sodium tolerable upper intake level of 2.3 g/day was used for all adult dietary reference intake age, sex, and life-stage groups [19]. Furthermore, according to the World Health Organization, eleven patients received 10 g of NaCMC daily for six months without complaints [20]. Moreover, adult patients have been treated for more than a year (>52 weeks) with daily oral doses of 2–6 g of NaCMC as a laxative without significant side effects [20]. For HEC oral ingestion per day in humans, HEC was administered in a dose of 1.0–1.5 g three times a day for at least 2 months without signs of toxicity [20]. Finally, for citric acid, there is no tolerable upper daily intake limit. It depends on acceptability of persons, e.g., a whole lemon (85 g) contains about 3 g of citric acid. Therefore, all these food grade materials are within the safe consumption level of adults.

### 4.5. Limitations of the Current Study

This paper reports on ongoing developments of the EndoXpand capsule and hence, there are many limitations at this stage. Limitations of this study are that we have not yet tested the cytotoxicity of the synthetic expandable material of the EndoXpand and developed a stimulus-responsiveness on-demand shrinkage outer membrane made by hydrogels that are supposed to achieve the triggerable EndoXpand capsules.

Another limitation is that we have not exposed fully swollen EndoXpand in SGF to cyclic compression as would occur in the antrum of the stomach using a mechanical testing device and have not yet developed a large species *in vivo* model, e.g., to visualize endoscopically or by ultrasonography the swelling and degradation of EndoXpand in the stomach after ingestion. The animals must be monitored for any evidence of tissue damage including vomiting, reduced fecal output, lethargy, abdominal distension, and mucosal redness and inflammation. A perfect large animal model does not exist for human application. A potential limitation of *in vivo* porcine models is that the gastric compression force in pigs is lower than that of humans [21,22]. Furthermore, pigs have slower gastric emptying than humans [20,23]. Before successful translation to humans, *in vivo* testing may be required in other large animal species such as dogs [24]. Lastly, whether varying occupied stomach volume by expandable EndoXpand capsules will avoid accommodation effects of the gastric cavity is yet uncertain. This clearly needs validation.

## 5. Conclusions

A novel bionics EndoXpand capsule to induce weight loss has been designed and bench tested in this study. It includes synthesized cellulose-based hydrogel particles as the core expandable materials, collagen casing as the outer membrane, and gelatin capsule shells. The expectation is that the volume occupied by the capsule in the stomach increases satiety. Weight loss can be controlled by changing the number of ingested capsules over a time period and their degradation time. In this way, we believe that accommodation effects can be avoided. The development of the ingestible capsule is intended to provide new design ideas for future weight-loss treatments. However, the research is still in a primary lab-based stage but ready for translation. Future work must explore further the properties and performance of EndoXpand capsules including safety assessment, development of triggerable outer membrane, and *in vivo* experimental studies.

## Data Availability

The data are available upon request to the corresponding author.
